# Virtual twins and the future of human developmental biology

**DOI:** 10.1038/s41540-026-00771-7

**Published:** 2026-06-23

**Authors:** Maria Abou Chakra, Nika Shakiba

**Affiliations:** 1 The Donnelly Centre, University of Toronto, Toronto, ON Canada; 2 School of Biomedical Engineering, University of British Columbia, Vancouver, BC Canada; 3 Premium Research Institute for Human Metaverse Medicine (PRIMe), The University of Osaka, Osaka, Japan

**Keywords:** Biological techniques, Biotechnology, Developmental biology, Stem cells

## Abstract

Human development begins with a zygote, yet how this cell forms complex tissues remains a mystery. Progress has been limited by ethical constraints and scarce biological materials. Advances in stem-cell models and virtual twins are now transforming research, healthcare, and our understanding of development. Collaboration among scientists, ethicists, and policymakers is essential to responsibly advance these tools. The Virtual Human Development consortium will bridge experts to co‑develop a reliable simulation platform.

## Introduction

Our bodies begin their journey as a single cell, called the zygote. The tiny zygote is formed when the egg and sperm meet, later giving rise to every type of cell in our bodies. Despite our growing understanding, this process remains one of the greatest puzzles in developmental biology: how does a single cell orchestrate the formation of something as complex as a human being? For decades, probing human development has been limited by scarce biological material and ethical concerns, creating barriers to deeper exploration. However, the emergence of technologies that enable us to see, quantify and store information about individual cells are improving at unprecedented rates, and these advancements have changed how we study these early stages^1^. We can now generate self-organized structures that mimic early human development, such as stem cell models^2,3^. Although these in vitro model systems are not identical to the human body, they can provide valuable insights that help us develop new regenerative therapies.

Concurrent with advances in stem cell models, computational technology has rapidly increased in speed and efficiency, paving the way for theoretical models of biology to catalyze groundbreaking shifts. These models provide a new perspective on the complex processes that shape human development, enabling us to overcome some of the traditional obstacles in the field. In silico models are advancing our understanding of developmental processes, such as cell behaviors^4–8^, morphogenesis^9–11^, and other complex structures^5,6,12–14^.

However, it is now time to take these models to the next level by integrating these theoretical models with cutting-edge stem cell models. By intersecting theory and experiments, we can catalyze groundbreaking advancements in “virtual twins”, in silico models^15^ that simulate parts of our bodies to predict how they function and when function is hampered by diseases, such as cancer^16^, immune^17,18^ or cardiovascular diseases^19,20^. The integration of models and data has accelerated discoveries about the biological rules governing human development. The next step is to establish a direct framework that creates a feedback loop among experts using in silico models and in vitro systems.

### Where do we start?

A successful virtual human twin (VHT) of development requires the integration of scientific fields. As more VHT projects emerge, we anticipate a paradigm shift from fields that typically work in silos to collaborative mergers where success is dependent on sharing and converging knowledge. This approach will change our scientific thinking; it is neither about the macroscale nor the mesoscale, nor about a reductionist view that has provided many clues and information over the years^6,10^. Rather, we should focus on connectivity and working across scales and perspectives—a multiscale (molecular-to-organ), integrative approach. VHTs are a promising tool to revolutionize healthcare procedures^21–27^. However, we believe that their success also demands transformation of the scientific process itself, starting with the integration of theory and experiments, in silico methods, scientific disciplines and policies.

### Integration of theory and experiments

Bridging experimental biology with theoretical modeling will advance our understanding of human development, disease mechanisms, and personalized therapies through improved modeling and simulation. Experts in different domains (such as computational biology, developmental biology, physics and engineering) approach the same fundamental developmental questions using diverse methods and perspectives. To advance integrative modeling, researchers must share their knowledge and ideas, reach consensus on foundational assumptions that are important for early development, identify sharing standards, and develop strategies to integrate established models.

Since communication is the first step, and is currently underway, the Virtual Human Development (VHD) consortium (www.virtualhumandevelopment.org) hosts the Bridging Theory and Experiment speakers series, discussing topics from both experimental and theoretical perspectives. Furthermore, examples such as the Virtual Physiological Human (VPH) Society for In Silico Medicine (vph-society.org) and the Integrative Multiscale Open Biology Community (https://sites.google.com/view/imobio/home) aim to unite all initiatives focused on developing human-driven virtual models across application areas. These communities are building essential infrastructure critical for field unification.

### Integration of in silico methods

In silico models are developed using mathematical and computational tools to capture a broad range of biological phenomena and systems, spanning the molecular, cellular and organ scales. Experiments are conducted virtually to answer a specific scientific or clinical question. Current in silico systems range from interpretable white-box approaches (equation-based or rule-based) to uninterpretable black-box approaches (deep neural networks). All of these models aim to interpret biological, chemical, and physical cues and responses in our bodies using algorithms that have the potential to predict how the system will behave under normal and abnormal conditions. The difference lies in the type of information that is provided. White-box models explicitly capture a phenomenon to explore and predict the system’s behavior. These models can capture multiscale phenomena, and the level of information generated depends on the resolution of the approaches used^28–30^. White box methods include differential equations, agent-based models, boolean or rule-based networks and tissue mechanics models (finite elements). Black-box models, traditionally statistical, now leverage machine learning and deep learning methods such as deep neural networks, random forests to recognize complex patterns in large datasets^31^.

However, siloed approaches limit progress. Hybrid gray-box models can combine mechanistic white-box models with data-driven black-box approaches (e.g., as deep learning and neural networks to identify patterns in data^32,33^). We think that hybrid models will provide interpretability and flexibility. Their combination can accelerate a crucial step in model development, which is encoding patterns analytically while preserving biological interpretability. For instance, theoreticians currently spend a substantial amount of time deriving the equations and rules that make the models biologically meaningful; emerging methods can now reduce discovery time by learning equations from data^34,35^. Other examples use neural networks to identify patterns in data to guide mechanistic models^32,33^. These steps are important for early development due to the scarcity and limited access to data.

While these approaches are just beginning to emerge, we are far away from true integration due to mismatched interfaces and disciplinary silos. Furthermore, hybrid models rarely produce a final equation or pattern without human curation. Thus, proper integration of approaches will require cross-disciplinary training and bidirectional collaborative efforts between experts.

### Integration of scientific disciplines: in silico and in vitro systems

Integrating lab-grown tissues (e.g., embryoids and organoids) with in silico modeling is a key step in developing VHTs that promise to revolutionize healthcare; by harnessing patient-specific data, VHTs enable expert researchers to test treatments without costly patient cohorts, to study diseases, and to optimize interventions for each unique patient, all in a safe and controlled environment^24,25,36,37^. Currently, the development of virtual organ models is in progress, with hopes that they may improve our understanding of organ function, accelerate recovery processes^38,39^, and even refine transplant procedures^40^. However, the possibilities do not stop with individual organs—researchers are working towards creating complete virtual human bodies^16^. This would be a monumental leap forward, offering insight into early human biology that is currently unattainable due to ethical and technological limitations in accessing and manipulating human tissues in labs.

With a fully developed embryo simulator, we can fill the gaps in our knowledge about our own development: how the tiny zygote builds our bodies. We can also make predictions about cell behavior and decisions in developmental systems we grow ex vivo, using the simulator to guide the engineering of cells and tissues on demand for therapies. Accessing the black-box of early development outside of ethical boundaries is a key advantage. However, once the baseline predictions are established and validated, simulators must model perturbation responses (e.g., pharmacological^41^ or toxicological^42,43^) to predict disease and therapeutic outcomes.

This vision requires bidirectional experimental-to-theory integration: in vitro systems should generate quantitative data that helps to form and refine simulations, while in silico predictions should guide experimental design through rapid rounds of hypothesis generation and testing. This iterative loop will transform siloed research into synergistic discovery and ensure the development of a reliable VHT system.

### Integration of policies: ethics and governance

While in silico systems offer many promising benefits, such as reducing animal research, aiding early disease detection, and providing risk assessment of invasive treatments, they also introduce ethical, technical and social risks. For instance, although technology may increase success rates and reduce treatment costs, it can also amplify inequities. On one hand, VHTs may increase access to healthcare information in rural and remote communities, owing to their computer-based nature. On the other hand, the ability to create patient-specific models depends on patient medical data and may be bottlenecked by access to medical testing or a patient’s proximity to laboratories for sample retrieval and analysis. To avoid some of these risks, clear and enforceable policies must govern data generation, sharing, storage and usage. Such policies need to extend outside of data management by including bias mitigation. Biases need to be treated as a continuous priority for the development and deployment of the systems. For example, models trained on limited human data will be susceptible to social and racial biases. To address this, we need concrete strategies that ensure human diversity is captured, such as regular dataset audits or applying established fairness metrics across all scales and developmental stages.

While improved data practices and bias mitigation strategies are essential, they are not sufficient on their own. The final decision or interpretation of the system should involve a human expert decision-maker. This human-in-the-loop approach^44^ is important to ensure that ethical judgment and accountability is maintained in decision-making; For example, EDITH is a project driven by VPH society and aims to provide a roadmap for digital twins in medicine (https://vph-society.org/project/edith/). A parallel document is needed for early developmental systems. As our methods advance, we may be able to develop genetic interventions that prevent the early onset of childhood diseases. Therefore, it is important to assess how we should use this knowledge ethically^45–47^. While the digital era can help mitigate the risk of diseases, it also opens up ethical debates around the terms and conditions under which we should intervene in human health^48^. Together, ethics and governance need to form a robust and responsible framework that defines ownership, control, and accountability across all stages of the use and development of reliable in silico systems.

### The future of early development with VHTs

The virtual twins of today are reshaping and reconfiguring how teams collaborate, having impacts that extend beyond the scientific discovery they accomplish. Diversity is crucial, with clinicians, experimentalists, and theoreticians working side by side. A variety of experts and tools are needed to conduct the best studies. Infrastructure should be modular to accommodate growth and change. Our simulators need to evolve and improve as our knowledge base expands. For example, the mission of the VHD consortium is to produce a state-of-the-art computer-based simulator of human embryonic development by bridging various disciplines across a global community of scholars. An area of anticipated early clinical impact for VHD is in the treatment of developmental diseases. Congenital diseases (such as heart defects, lung pulmonary malformations or neurodevelopmental disorders) that can be detected *in utero* and corrected after birth would benefit from non-intrusive simulators. For instance, VHD has the potential to be used to simulate different embryo outcomes based on measured changes during pregnancy. Using these models, researchers can run virtual trials of new therapeutics or drug treatments. These models can be used to inform the healthcare team in planning the best treatment strategies for each patient. This proactive approach could ensure better health outcomes for managing congenital diseases, paving the way for transformative advancements in medical treatments, personalized care, and regenerative medicine.

**Fig. 1:**
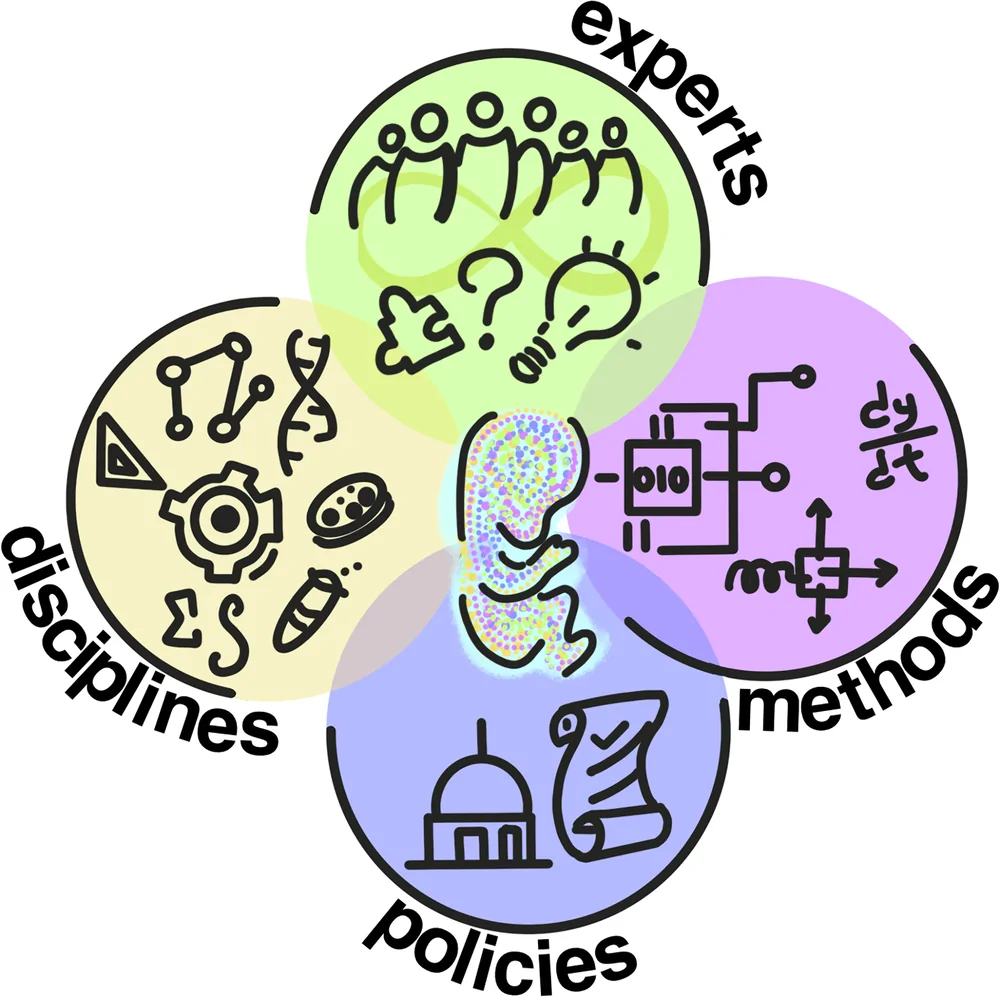
Towards the vision of a virtual model of human development. The vision requires integration of experts in theory and experiments across disciplines, methods and policies. These are the first steps that will improve biological predictions and shift the scientific process.

We stand at the edge of a future where VHT technologies promise to unlock the deepest mysteries of our biology, which were once considered inaccessible. This is not just a leap forward but a potential shift in how we understand and use this knowledge in medical therapies. Integrating VHTs with data-driven and experimental knowledge is only the beginning. VHTs offer the potential for personalized treatments, improved health outcomes, and reduced risks. However, as we push the boundaries to a whole new realm by modeling a human embryo—our responsibility grows^45–47^. Our path needs to be carefully navigated with transparency, ethical rigor^49^ and collaboration. Achieving robust, reliable virtual systems capable of simulating and predicting early human development will require shared standards, systematic validation frameworks and collaborations among domain experts. We call upon policymakers, clinicians, and ethicists to join the global VHD community, a network of 40+ pioneering researchers, to shape this future together. The future demands that we not only harness the potential of VHTs but also do so with forethought and care, ensuring that this innovation serves the highest public good Fig. 1.

## Data Availability

No datasets were generated or analysed during the current study.
